# Drug resistance and epidemiology characteristics of multidrug-resistant tuberculosis patients in 17 provinces of China

**DOI:** 10.1371/journal.pone.0225361

**Published:** 2019-11-21

**Authors:** Zhenhui Lu, Wenhan Jiang, Jing Zhang, Henry S. Lynn, Yue Chen, Shaoyan Zhang, Zifeng Ma, Peihua Geng, Xiaoyan Guo, Huiyong Zhang, Zhijie Zhang

**Affiliations:** 1 Department of Respiratory, Longhua Hospital Shanghai University of Traditional Chinese Medicine, Shanghai, People’s Republic of China; 2 Department of Epidemiology and Biostatistics, School of Public Health, Fudan University, Xuhui District, Shanghai, People’s Republic of China; 3 School of Epidemiology, Public Health and Preventive Medicine, Faculty of Medicine, University of Ottawa, Ottawa, Ontario, Canada; The University of Hong Kong, CHINA

## Abstract

As China is one of high MDR-TB burden countries, it is important to determine the drug resistant pattern and clinical characteristics of multidrug resistant tuberculosis (MDR-TB). We conducted a comprehensive and nationwide study on MDR-TB in 17 provinces for the period from June 2009 to June 2015, and a total of 1154 cases of MDR-TB were finally investigated. The study sought to assess the clinical features and contrast drug susceptibility profiles of MDR-TB patients in China. Cavitary disease, young age, and long duration of TB disease among MDR-TB patients were important predictors. A high resistance proportion of first-line drugs was observed in Beijing, Shanghai and Tianjin. Resistant proportions of second-line anti-TB drugs in western region for amikacin, aminosalicylic acid, and levofloxacin were higher than eastern and central regions. High levels of drug resistance were seen in earlier cases (before 2011) and outpatients. We found high levels of resistance to 1st- and 2nd-line drugs in all settings, with considerable variabilities in terms of different Directly Observed Treatment Short Course (DOTS) programme, level of economic development(eastern, central and western regions) and patient source (inpatients and outpatients). Timely drug susceptibility testing (DST) and effective management are necessary to ensure an early detection of MDR-TB and its proper treatment.

## Introduction

Multidrug-resistant tuberculosis (MDR-TB) is caused by bacteria that are resistant to both isoniazid and rifampicin, the most effective anti-TB drugs, or more. MDR-TB presents a major public health concern in many countries and continues to threaten TB control[1, 2]. In 2017, there was an estimated 558 000 people developed rifampicin-resistant-TB, and of these, 82% had MDR-TB. Three countries accounted for almost half of the world’s cases of MDR-TB: India (24%), China (13%) and the Russian Federation (10%)[3]. An estimated 9.7% of people with MDR-TB have extensively drug-resistant TB (XDR-TB), which is defined as MDR-TB plus resistance to at least one fluoroquinolone and a second-line injectable agent. The treatment of MDR-TB requires extensive chemotherapy, a WHO recommended regimen in 2016 with at least four effective TB medicines during the intensive phase, including fluoroquinolones and second-line injections as core drugs, the total course of treatment is 18–24 months, and the intensive period is 6 months. In addition, second-line drugs (SLDs) are very expensive compared with the drugs for standard new TB treatment and can also cause a range of serious side effects[4–6] The treatment of XDR-TB is more complex. Even in developed regions, the success rate is low for the treatment of MDR/XDR-TB. China is one of the countries with a high burden of MDR-TB, and approximately 50% of MDR-TB cases worldwide occur in China and India. In 2007, 8.3% of TB patients had MDR-TB in China. It was estimated that about 110,000 new MDR-TB cases occurred every year, and among them 7.2% had XDR-TB. In 2010, a national survey estimated that MDR-TB accounted for 6.8% of total incident cases [7]. To reduce the incidence of drug-susceptible tuberculosis, it is important to have a better understanding of the occurrence and transmission patterns of disease and risk factors associated with MDR-TB. A number of recent studies have provided some information on socio-demographic and clinical features of patients with drug resistant TB from different geographical areas or provinces in China[8–10], and the results vary with study population and geographical area. In addition, up to 2010, nearly half of the patients diagnosed in the hospital system still had not been reported to the Centers for Disease Control(CDC). We conducted a nationwide hospital-based survey of MDR-TB patients covering 17 provinces of the country for the period from 2009 to 2015, to identify epidemiologic and geographic variations in MDR-TB and risk factors for drug resistance among MDR-TB cases.

## Methods and methods

### Study design

**Fig 1:**
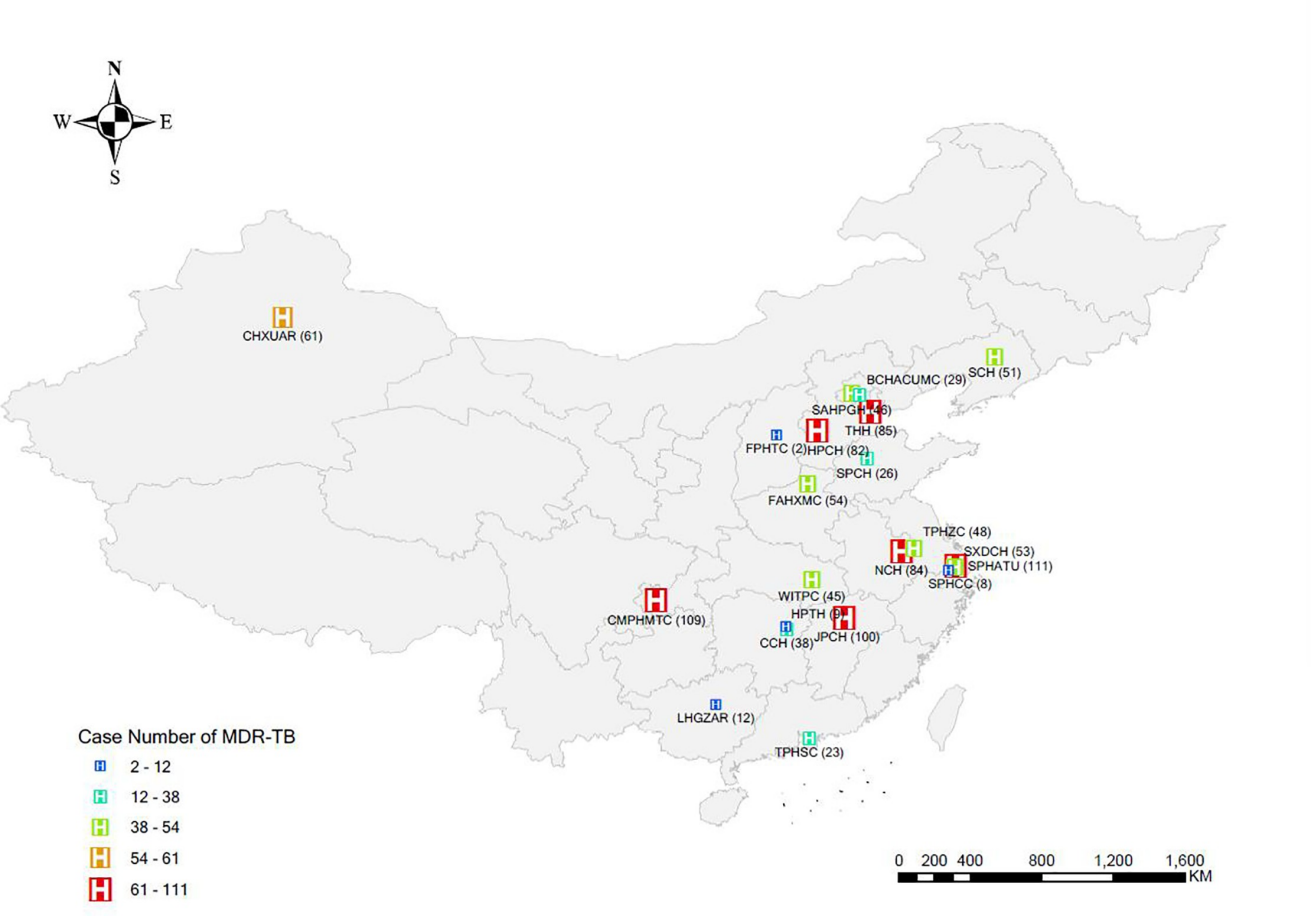
Distribution of the survey sites. Note(hospital): CHXUAR: Chest Hospital of Xinjiang Uygur Autonomous Region; SCH: Shenyang Chest Hospital; BCHACUMC: Beijing Chest Hospital Affiliated to Capital University of Medical Sciences; SAHPGH: The Second Affiliated Hospital of the PLA General Hospital; THH: Tianjin Haihe Hospital; FPHTC: The Fourth People 's Hospital of Taiyuan City; HPCH: Hebei Province Chest Hospital; SPCH: Shandong Province Chest Hospital; FAHXMC: The First Affiliated Hospital of Xinxiang Medical College; TPHZC: The Third People 's Hospital of Zhenjiang City; NCH: Nanjing Chest Hospital; SPHATU: Shanghai Pulmonary Hospital Affiliated to Tongji University; SXDCH: Shanghai Xuhui District Central Hospital; SPHCC: Shanghai Public Health Clinical Center; WITPC: Wuhan Institute of Tuberculosis Prevention and Control; CMPHMTC: Chongqing Municipal Public Health Medical Treatment Center; ZCCMH: Zigong City Chinese Medicine Hospital; CCH: Changsha Central Hospital; HPTH: Hunan Provincial Tuberculosis Hospital; JPCH: Jiangxi Provincial Chest Hospital; LHGZAR: Longtan Hospital of Guangxi Zhuang Autonomous Region; TPHSC: The Third People 's Hospital of Shenzhen City.

**Fig 2:**
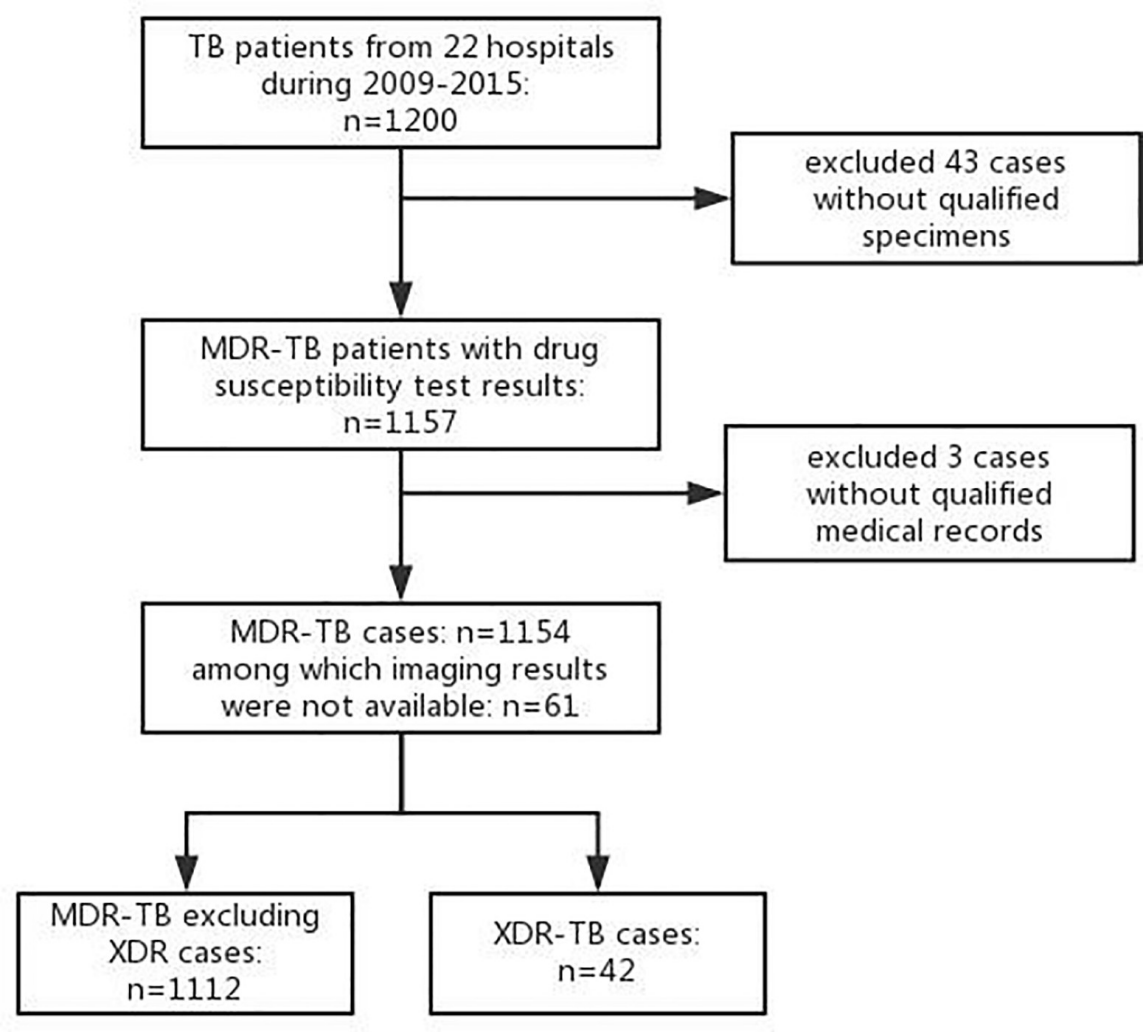
Study population of MDR-TB in 22 hospitals in 17 provinces of China.

The study was conducted in 22 tertiary care hospitals or specialized tuberculosis hospitals in 17 provinces of China (Fig 1). All MDR-TB patients diagnosed or treated in the 22 hospitals between the ages of 16 and 75 years with sputum culture–positive were included in the study. During 2009–2015, 1200 eligible tuberculosis patients were investigated and interviewed by a qualified physician in the study hospitals, and 46 were excluded because of unsatisfactory specimens (43) or incomplete medical records (3). Of the remaining 1154 patients, 1093 had imaging examination and 42 had XDR-TB (Fig 2).

### Sputum specimens and cultures

Sputum specimens were collected for culture testing at admission. If necessary, induced sputum was collected. Sputum specimens were digested and decontaminated using the N-acetyl-L- cysteine-sodium hydroxide method; the final sodium hydroxide concentration was 2%. All specimens were tested using the BACTEC-MGIT 960 automated system.

Cultures were prepared according to the manufacturer's instructions using a BACTEC-MGIT 960 automated system (Becton Dickinson Co., MD, U.S.A.). The positive culture organisms were tested using acid-fast staining (AFB) with Ziehl-Neelsen or Auramine O. The AFB-positive cultures were confirmed by the p-nitrobenzoic acid (PNB)[11].

### Drug susceptibility testing

Drug-susceptibility testing was performed at the clinical laboratory of each enrolled hospital. MDR-TB isolates underwent susceptibility testing with an automated BACTEC-MGIT 960 culture system using a BACTEC MGIT SIRE kit from Becton, Dickinson (Sparks, MD). A standard protocol for the BACTEC-MGIT 960 system was followed according to the manufacturer’s instructions[12]. When the growth control reached a growth unit (GU) value of 400, the GU values of drug-containing tubes were retrieved from the instrument by printing out a DST set report, and results were interpreted manually. If the GU of the drug-containing tube was more than 100, the results were defined as resistant, the results were considered susceptible if the GU was equal to or less than 100. Sensitivity results were determined by comparing the fluorescence in the drug-containing tube with the fluorescence in the growth control tube. The critical concentration of second-line drugs is levofloxacin 2.0ug/ml, amikacin 1ug/ml, and para-aminosalicylic acid 4.0ug/ml, respectively[13].

### Socio-demographic and clinical characteristics

Basic socio-demographics and clinical characteristics of patients were obtained from well-designed questionnaires and medical records.

### Imagological diagnosis

Chest computed tomography (CT) was performed on each patient at the start of enrollment. Results were read by senior radiologists who were affiliated with the enrolled hospitals. In case of any discrepancy, results would be confirmed by a third evaluator.

### Data preprocessing

MDR-TB cases were divided into two groups: new cases, and previously treated cases. A new case was defined as a patient who has not been diagnosed as TB before or had any prior anti-TB treatment for 30 days or less, a previously treated case was defined as a patient who had been diagnosed for TB for more than 30 days before he or she was diagnosed as MDR-TB.

According to the level of economic development, we divided these provinces into three regions: eastern region includes Beijing, Fujian, Guangdong, Hainan, Heibei, Jiangsu, Liaoning, Shandong, Shanghai, Tianjin, and Zhejiang; Central region includes Anhui, Heilongjiang, Henan, Hunan, Hubei, Jiangxi, Jilin, and Shanxi; Western region includes Chongqing, Gansu, Guangxi, Guizhou, Neimeng, Ningxia, Qinghai, Shaanxi, Sichuan, Tibet, Xinjiang, and Yunnan.

We also used a similar approach proposed by Wang[14] to classify the provinces into 3 groups according to the DOTS strategy: Group 1 included Beijing, Shanghai, and Tianjin and these three municipalities had TB control programmes similar to DOTS and higher economic development for more than 30 years; Group 2 included Chongqing, Gansu, Guangdong, Hainan, Hebei, Heilongjiang, Hubei, Hunan, Liaoning, Ningxia, Shandong, Sichuan, and Xinjiang, and these provinces implemented DOTS in 1990s (DOTS1990); Groups 3 included Anhui, Fujian, Guangxi, Guizhou, Henan, Jiangsu, Jiangxi, Jilin, Neimeng, Qinghai, Shaanxi, Shanxi, Tibet, Yunnan, and Zhejiang, and these provinces implemented DOTS after 2000 (DOTS2000).

We also classified the cases into two time periods: 2009–2010 and 2011–2015. One tuberculosis-10-year plan ended in 2010, and a new tuberculosis-10-year plan started in 2010.

### Statistics

Data were presented as mean ± standard deviation (SD) for quantitative variables and frequency and percentages for qualitative variables. Mantel-Haenszel chi-square test was used to evaluate univariate risk factors associated with drug resistance patterns. Unconditional logistic regression was used to investigate drug resistant patterns among groups with different epidemiological characteristics and clinical features by estimating adjusted odds ratios (ORs) and 95% confidence intervals (CIs). Variables with a p value of less than 0.15 in the univariate analysis and other relevant variables were included in unconditional logistic regression models. Statistical tests were two-tailed with the significance level set at α < 0.05. Data were analyzed using SAS 9.4 (SAS Institute Inc., Cary, NC, USA).

### Study approval

Ethical approval for this study was obtained from the Medical Ethics Committee of Longhua Hospital Affiliated to Shanghai University of Traditional Chinese Medicine. Written informed consent was obtained from each participant.

## Results

### Distribution and general characteristics of the study population

During the period from April 2009 to December 2015, we investigated 1154 MDR-TB patients from 22 hospitals in China. Among them, 622 (53.90%) were inpatients; none was HIV positive. 672 (58.2%) of patients were investigated in 2011 or later. The median age was 38 years (range 16–75), and 757 cases (65.6%) were 45 years old or less. Of the patients 777 were male (67.3%). Moreover, 219 (19.0%) cases are farmers. (Table 1)

**Table 1:** Social demographic characteristics of subjects.

Variables	East	Central	West	Combine
	673	265	216	1154
Period	673	265	216	1154
Before 2011	302 (44.87%)	137 (51.70%)	43 (19.91%)	482 (41.77%)
In 2011 or later	371 (55.13%)	128 (48.30%)	173 (80.09%)	672 (58.23%)
Gender	673	265	216	1154
Male	465 (69.09%)	179 (67.55%)	133 (61.57%)	777 (67.33%)
Female	208 (30.91%)	86 (32.45%)	83 (38.43%)	377 (32.67%)
Age	673	265	216	1154
Mean ± SD	39.0± 12.89	38.2± 13.17	37.6± 12.94	38.5± 12.96
Median, Range	38.0, 58.0	37.0, 52.0	36.0, 52.0	38.0, 59.0
Min, Max	17.0, 75.0	17.0, 69.0	16.0, 68.0	16.0, 75.0
Age group	673	265	216	1154
16–30	210 (31.20%)	86 (32.45%)	78 (36.11%)	374 (32.41%)
31–45	224 (33.28%)	87 (32.83%)	72 (33.33%)	383 (33.19%)
45–60	194 (28.83%)	77 (29.06%)	51 (23.61%)	322 (27.90%)
>60	45 (6.69%)	15 (5.66%)	15 (6.94%)	75 (6.50%)
Marital status	673	265	216	1154
Unmarried	172 (25.56%)	73 (27.55%)	70 (32.41%)	315 (27.30%)
Married	501 (74.44%)	192 (72.45%)	146 (67.59%)	839 (72.70%)
Occupation	673	265	216	1154
Farmer	141 (20.95%)	38 (14.34%)	40 (18.52%)	219 (18.98%)
Other	532 (79.05%)	227 (85.66%)	176 (81.48%)	935 (81.02%)
Ethnic	673	265	216	1154
Han	649 (96.43%)	263 (99.25%)	180 (83.33%)	1092 (94.63%)
Other	24 (3.57%)	2 (0.75%)	36 (16.67%)	62 (5.37%)
Case source	673	265	216	1154
Outpatient	329 (48.89%)	128 (48.30%)	75 (34.72%)	532 (46.10%)
Inpatient	344 (51.11%)	137 (51.70%)	141 (65.28%)	622 (53.90%)
BMI	673	265	216	1154
Mean ± SD	20.4± 3.27	19.9± 2.97	20.3± 3.06	20.3± 3.17
Median, Range	20.2, 24.2	19.5, 17.3	20.0, 18.8	20.0, 24.2
Min, Max	9.8, 34.0	12.3, 29.7	13.5, 32.3	9.8, 34.0

### Clinical characteristics of subjects

366 (31.7%) of 1154 had less than 1 year of TB treatment, and 376 (32.6%) experienced more than 3 years of treatment. 363 (34.3%) of 1060 who were previously treated were diagnosed as MDR-TB within one year of tuberculosis, 251(23.7%) within three years of tuberculosis, and 446(42.1%) after more than 3 years of tuberculosis. Median drug resistant number was 1 (range: 0–5), which means at least half of the MDR-TB patients included in this study had one or more additional drug resistance besides isoniazid and rifampin resistance. Only 94 (8.2%) of the cases were new cases (Table 2).

**Table 2:** Clinical characteristics of subjects.

Variables	East	Central	West	Combine
	673	265	216	1154
Tb duration (month)	673	265	216	1154
< = 12 months	209 (31.05%)	78 (29.43%)	79 (36.57%)	366 (31.72%)
12–60 months	244 (36.26%)	95 (35.85%)	73 (33.80%)	412 (35.70%)
>60 months	220 (32.69%)	92 (34.72%)	64 (29.63%)	376 (32.58%)
Time between TB & MDR TB (month) *				
< = 12 months	203 (33.12%)	89 (35.32%)	71 (36.41%)	363 (34.25%)
12–36 months	148 (24.14%)	54 (21.43%)	49 (25.13%)	251 (23.68%)
>36 months	262 (42.74%)	109 (43.25%)	75 (38.46%)	446 (42.08%)
Drug resistance number	673	265	216	1154
Mean ± SD	1.1± 1.09	1.0± 1.14	1.4± 1.13	1.2± 1.11
Median, Range	1.0, 5.0	1.0, 5.0	1.0, 5.0	1.0, 5.0
Min, Max	0.0, 5.0	0.0, 5.0	0.0, 5.0	0.0, 5.0
MDR within 30 days after TB	673	265	216	1154
< = 30 days	60 (8.92%)	13 (4.91%)	21 (9.72%)	94 (8.15%)
>30 days	613 (91.08%)	252 (95.09%)	195 (90.28%)	1060 (91.85%)

### Imaging characteristics of subjects

The median number of lobes with lesion was 4 (range 0–6). Among the 1154 patients, 1093 had complete chest radiographic findings. 762(69.7%) cases had at least one cavity and 241 (22.1%) cases had three or more cavities with a median cavity diameter being 1.00 cm (range 0–9) for all the patients (Table 3).

**Table 3:** Imaging characteristics of subjects.

Variables	East	Central	West	Combine
	673	265	216	1154
Lopes with lesion	639	254	200	1093
Mean ± SD	3.8± 1.70	3.6± 1.72	3.5± 1.74	3.7± 1.71
Median, Range	4.0, 6.0	4.0, 6.0	4.0, 6.0	4.0, 6.0
Min, Max	0.0, 6.0	0.0, 6.0	0.0, 6.0	0.0, 6.0
Any cavity exists?	639	254	200	1093
No	210 (32.86%)	57 (22.44%)	41 (20.50%)	308 (28.18%)
Yes	429 (67.14%)	197 (77.56%)	159 (79.50%)	785 (71.82%)
No. of cavities	639	254	200	1093
0	215 (33.65%)	62 (24.41%)	54 (27.00%)	331 (30.28%)
1	202 (31.61%)	75 (29.53%)	61 (30.50%)	338 (30.92%)
2	105 (16.43%)	43 (16.93%)	35 (17.50%)	183 (16.74%)
> = 3	117 (18.31%)	74 (29.13%)	50 (25.00%)	241 (22.05%)
Cavity diameter (cm)	639	254	200	1093
Mean ± SD	1.5± 1.69	1.7± 1.58	1.3± 1.53	1.5± 1.64
Median, Range	1.0, 9.0	1.5, 9.0	0.5, 8.0	1.0, 9.0
Min, Max	0.0, 9.0	0.0, 9.0	0.0, 8.0	0.0, 9.0

### Drug-resistant pattern of univariate analysis

Generally, the anti-TB drug resistance was higher for the first-line drugs (FLDs) than the SLDs. Specifically, for FLDs, 655(56.8%) of the total cases were resistant to streptomycin, followed by 362(31.4%) ethambutol resistance. For SLDs, levofloxacin resistant cases were 160(13.9%), followed by 87(7.5%) amikacin resistance and 77(6.7%) aminosalicylic acid resistance. And there were 42 (3.6%) cases who were confirmed as XDR-TB.

The drug-resistant patterns were different across the three DOTS areas. The ratio of ethambutol in Group 1 (Beijing, Shanghai and Tianjin) was 44.7%, whereas 26.7% in Group 2 (DOTS 1990 areas) and 24.5% in Group 3 (DOTS 2000 areas), and there was a significant difference among the three groups. Streptomycin predominated in MDR-TB cases, accounting for 62.1% in Group 1, 56.0% in Group 2, and 52.2% in Group 3. The drug resistant rate was also significantly different for levofloxacin among the three groups. Among the 502 MDR cases from DOTS1990 area, 134 (26.7%) were resistant to at least one SLD, which was significantly higher than the other two areas.

Compared with eastern and central regions, western region had a higher SLD resistance rate for all the three SLDs. In western region, the resistant proportion was 9.2% for amikacin, 10.1% for aminosalicylic acid, and 26.9% levofloxacin respectively.

Compared the time period before 2011 to the period of 2011 or after, the resistant proportion was significantly higher for the following 4 drugs: ethambutol (36.5% vs 27.7%), streptomycin resistance (63.7% vs 51.8%), amikacin (10.6% vs 5.4%), and aminosalicylic acid (9.8% vs 4.5%). The resistant proportion was also higher for at least one SLD and XDR before 2011 compared with 2011 or after.

As for FLDs, streptomycin show the highest resistant proportion in both outpatients and inpatients, and followed by ethambutol. As for SLDs, the proportion of levofloxacin resistance was the highest, and followed by amikacin and aminosalicylic acid resistance. Outpatients had a higher proportion of resistance than inpatients for ethambutol, streptomycin, aminosalicylic acid and levofloxacin. 130 (24.4%) outpatients were resistant to at least one SLD. On the other hand, 109 (17.5%) inpatients were resistant to at least one SLD.

Patients with cavitary disease had a higher proportion of drug resistance than those without. More detailed information on patient drug susceptibility pattern is shown in Table 4.

**Table 4:** Univariate risk factors associated with drug resistance pattern.

Variables		Ethambutol	Streptomycin	Amikacin	Aminosalicylic acid	Levofloxacin	At least one SLD	XDR-TB
		362 (31.37%)	655 (56.76%)	87 (7.54%)	77 (6.67%)	160 (13.86%)	239 (20.71%)	42 (3.64%)
DOTS area (P value)		39.70 (< .001)*	6.72(0.035)*	1.22(0.542)	6.35(0.042)*	32.21 (< .001)*	22.09 (< .001)*	1.37(0.504)
DOTS 1990(Group 2)	502	134 (26.69%)	281 (55.98%)	35 (6.97%)	41 (8.17%)	102 (20.32%)	134 (26.69%)	21 (4.18%)
DOTS 2000(Group 3)	314	77 (24.52%)	164 (52.23%)	22 (7.01%)	23 (7.32%)	23 (7.32%)	42 (13.38%)	12 (3.82%)
Beijing, Shanghai, Tianjin(Group 1)	338	151 (44.67%)	210 (62.13%)	30 (8.88%)	13 (3.85%)	35 (10.36%)	63 (18.64%)	9 (2.66%)
Region (P value)		8.03(0.018)*	1.97(0.373)	1.82(0.402)	11.80(0.003)*	43.64 (< .001)*	38.52 (< .001)*	7.54(0.023)*
East	673	231 (34.32%)	372 (55.27%)	47 (6.98%)	31 (4.61%)	85 (12.63%)	129 (19.17%)	17 (2.53%)
Central	265	66 (24.91%)	152 (57.36%)	19 (7.17%)	23 (8.68%)	17 (6.42%)	34 (12.83%)	11 (4.15%)
West	216	65 (30.09%)	131 (60.65%)	21 (9.72%)	23 (10.65%)	58 (26.85%)	76 (35.19%)	14 (6.48%)
Period (P value)		10.17(0.001)*	16.20 (< .001)*	10.98 (< .001)*	12.59 (< .001)*	0.30(0.584)	5.67(0.017)*	5.64(0.018)*
Before 2011	482	176 (36.51%)	307 (63.69%)	51 (10.58%)	47 (9.75%)	70 (14.52%)	116 (24.07%)	25 (5.19%)
In 2011 or later	672	186 (27.68%)	348 (51.79%)	36 (5.36%)	30 (4.46%)	90 (13.39%)	123 (18.30%)	17 (2.53%)
Gender (P value)		0.83(0.362)	0.26(0.611)	4.15(0.042)*	0.51(0.474)	1.49(0.222)	1.50(0.220)	5.95(0.015)*
Male	777	237 (30.50%)	437 (56.24%)	50 (6.44%)	49 (6.31%)	101 (13.00%)	153 (19.69%)	21 (2.70%)
Female	377	125 (33.16%)	218 (57.82%)	37 (9.81%)	28 (7.43%)	59 (15.65%)	86 (22.81%)	21 (5.57%)
Age group (P value)		2.03(0.565)	0.92(0.820)	3.29(0.349)	3.78(0.287)	6.87(0.076)	7.73(0.052)	3.35(0.340)
16–30	374	107 (28.61%)	214 (57.22%)	27 (7.22%)	25 (6.68%)	54 (14.44%)	82 (21.93%)	12 (3.21%)
31–45	383	127 (33.16%)	223 (58.22%)	36 (9.40%)	30 (7.83%)	64 (16.71%)	92 (24.02%)	19 (4.96%)
45–60	322	104 (32.30%)	177 (54.97%)	20 (6.21%)	15 (4.66%)	32 (9.94%)	51 (15.84%)	8 (2.48%)
>60	75	24 (32.00%)	41 (54.67%)	4 (5.33%)	7 (9.33%)	10 (13.33%)	14 (18.67%)	3 (4.00%)
Case source (P value)		13.72 (< .001)*	8.90(0.003)*	3.11(0.078)	6.17(0.013)*	5.11(0.024)*	8.34(0.004)*	4.38(0.036)*
Outpatient	532	196 (36.84%)	327 (61.47%)	48 (9.02%)	46 (8.65%)	87 (16.35%)	130 (24.44%)	26 (4.89%)
Inpatient	622	166 (26.69%)	328 (52.73%)	39 (6.27%)	31 (4.98%)	73 (11.74%)	109 (17.52%)	16 (2.57%)
Tb duration (P value)		4.17(0.125)	1.64(0.441)	2.95(0.229)	1.76(0.416)	7.62(0.022)*	2.97(0.226)	3.94(0.139)
< = 12 months	366	113 (30.87%)	206 (56.28%)	22 (6.01%)	28 (7.65%)	36 (9.84%)	67 (18.31%)	8 (2.19%)
12–60 months	412	117 (28.40%)	226 (54.85%)	30 (7.28%)	29 (7.04%)	62 (15.05%)	84 (20.39%)	20 (4.85%)
>60 months	376	132 (35.11%)	223 (59.31%)	35 (9.31%)	20 (5.32%)	62 (16.49%)	88 (23.40%)	14 (3.72%)
MDR within 30 days after TB (P value)		0.34(0.560)	0.26(0.609)	0.72(0.395)	0.55(0.456)	1.58(0.209)	0.85(0.357)	0.67(0.414)
< = 30 days	94	32 (34.04%)	51 (54.26%)	5 (5.32%)	8 (8.51%)	9 (9.57%)	16 (17.02%)	2 (2.13%)
>30 days	1060	330 (31.13%)	604 (56.98%)	82 (7.74%)	69 (6.51%)	151 (14.25%)	223 (21.04%)	40 (3.77%)
Any cavity exists? (P value)		0.77(0.382)	0.60(0.440)	0.24(0.624)	4.68(0.031)*	2.47(0.116)	5.19(0.023)*	0.66(0.416)
No	308	92 (29.87%)	171 (55.52%)	22 (7.14%)	13 (4.22%)	35 (11.36%)	51 (16.56%)	9 (2.92%)
Yes	785	256 (32.61%)	456 (58.09%)	63 (8.03%)	62 (7.90%)	118 (15.03%)	179 (22.80%)	31 (3.95%)

DOTS = directly observed treatment short course; SLD = second-line drugs; XDR = extensively drug-resistant.

* Mantel-Haenszel chi-square test

### Multivariate regression analysis of risk factors

**Fig 3:**
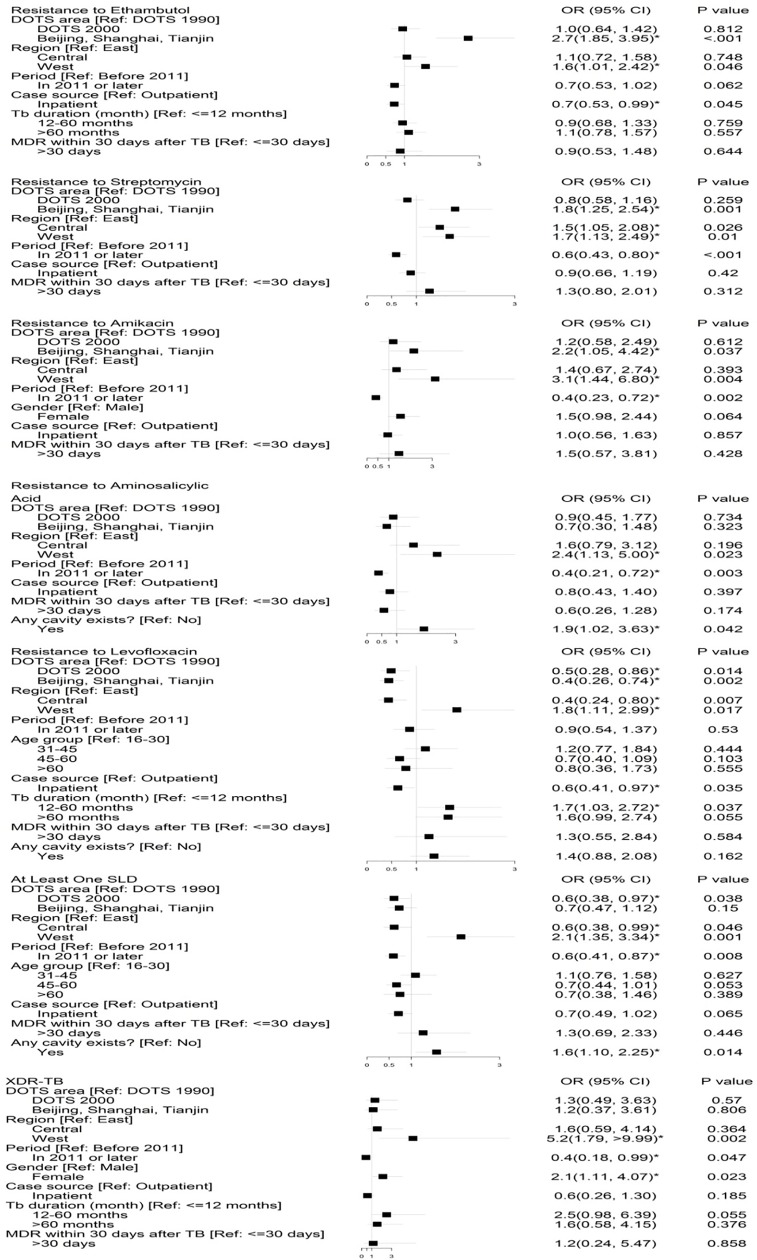
Multivariate logistic regression analysis of the resistant pattern.

DOTS area and time period were significantly associated with drug resistance. Group 1 (Beijing, Shanghai and Tianjin) had an increased risk of resistance to FLDs as compared to Group 2 (DOTS1990 areas). Group 3 (DOTS2000 areas) had a reduced risk for the resistance to levofloxacin than Group 2 (adjusted OR = 0.5, 95% CI: 0.28, 0.86) and Group 1 (adjusted OR = 0.4, 95% CI: 0.26, 0.74). In terms of geographical region, western region had significant increased risks of resistance to all FLDs, SLDs and XDR when compared with other regions. The time period of 2011 or after was associated with higher risks of resistance to streptomycin, amikacin, aminosalicylic acid, at least one SLD resistance and XDR. Outpatients had a high risk of drug resistance in general for those with MDR, although statistical significance was only observed for the resistance to ethambutol and levofloxacin. The relationship between cavitary disease and high drug resistance to aminosalicylic acid and at least one SLD were statistically significant. No statistical differences in drug resistance were found between new cases and previously treated cases (Fig 3).

## Discussion

Our study of more than 1000 cases of MDR-TB from tertiary care hospitals or specialized tuberculosis hospital during 2009–2015 in 17 provinces and metropolitan cities in China showed that the mean age was 38.5 years, and patients aged 45 years or younger accounted for 65.6% of the total cases. The frequency of MDR-TB peaked in young adulthood and the age profile of MDR-TB was in agreement with other reports [15–17]. In another study analyzing treatment outcomes of patients with MDR-TB or XDR-TB in Shanghai, more than 80% of the 175 patients were less than 60 years old[16]. A retrospective study showed that MDR-TB was more frequent among patients aged 25 to 44 years in China[18]. It suggested that it is important to develop strategies for TB control according to age. In our study, the majority of MDR-TB patients (67.33%) were male, similar to the result from a study carried out in Germany [19] and those from most other countries [20]. The fifth national tuberculosis epidemiological survey in China in 2010 showed that men had a higher prevalence of TB than women in all age groups[7]. A high proportion of cavitary disease among MDR-TB patients was observed in our study. Cavitary disease has been associated with MDR-TB in previous research[18, 21]. Our study showed that 697 (65.8%) previously treated patients were diagnosed as MDR-TB after one year of tuberculosis. It indicated that most previously treated patients had a long course of TB. Prolonged length of treatment is one of the common risk factors that may promote drug resistance [22, 23]. Since routine DST is absent in most areas of China, it is hard to discover MDR-TB in early stages and adjust the therapeutic regimen in time.

For the first line anti-TB drug, the proportion of drug resistance of streptomycin (56.8%) among MDR cases in our study were close to that observed from a previous study in Beijing (51.9%)[21]. In 2010, a hospital based study in VietNam, 92% of 188 patients with MDR-TB were resistant to streptomycin. The high proportion of streptomycin resistant cases, especially a high level of resistance to streptomycin in Beijing, Shanghai, Tianjin, indicated that this drug was unlikely to benefit many patients. And this result is consistent with present WHO treatment strategy that streptomycin is not recommended for MDR-TB cases[24]. Levofloxacin is widely used for respiratory-tract and other infections, and fluoroquinolones are extensively used in people who are later diagnosed with tuberculosis, which might result in a high proportion of resistance to fluoroquinolones in patients with MDR-TB[25, 26]. Our survey showed that overall 20.7% MDR-TB patients were resistant to at least one SLD. This proportion was lower than those from previous reports from China (54.4%)[27] and India (44.8%)[28]. Given that some SLDs such as kanamycin and capreomycin were not tested in our study, the proportion of resistance to SLDs in our study might be underestimated.

To study the impact of treatment strategy, we compared the drug susceptibility profiles among different DOTS areas. And the results showed that the proportion of first-line drug resistance was higher in Beijing, Shanghai and Tianjin. Considering that a large proportion of the patients in Beijing, Shanghai and Tianjin were migrant population, and refractory and severe patients tended to seek treatment in large cities, this result was likely a consequence of highly selective source of cases[29].

Attention should be paid to the high proportion of anti-TB drugs resistance in western region. A previous study showed that western region had the highest prevalence of TB in China, and the ability to control the disease lagged behind the burden of TB[14, 30]. The high drug resistant rate might be partially due to low economic level and tuberculosis management in the region. It is difficult for a low household income family to cover high MDR treatment costs, which leads to a poor adherence to MDR-TB treatment, and causes the emergence of drug resistance[31]. In addition, many MDR-TB patients might not have access to adequate treatment of sufficient quality.

Multiple drug resistance cases existed in different periods with considerable variability. During 2009–2010, all the drug resistance proportions were higher than those during 2011–2015. The reduction in proportions of two first line drugs and two SLD was significant. The latest National Tuberculosis Control Program in China was covered from 2011 to 2015, which means a change point for TB control. The reduction could be attributed to a result of the rapid development of the TB control systems such as scale-up of the DOTS programme nationwide and improvement of public health facilities [32]. And the high drug resistance rate before 2011 was consistent with Chinese government’s commitment to detect 70% of all new smear-positive cases and successfully treat 85% of these cases. A series of policies of TB prevention and control was launched during that period, which might contribute to the high drug resistance detection rate.

There were some differences in drug resistance between outpatients and inpatients. In general, inpatients received better treatment management with a better treatment adherence. MDR-TB usually takes at least 18 months of treatment. Approximately half of TB outpatients had a poor compliance[33], and many outpatients stopped their medication when the situation improved[24]. No statistical differences were found regarding age, gender and new or previously treated cases in drug resistant patterns.

There are several limitations to our study. First, individuals younger than 15 years were not included in our study. Second, information on long-term treatment outcomes was not collected in this study. Long-term clinical efficacy in these patients needs to be investigated to further understand the recurrence of MDR. Third, HIV-infected tuberculosis patients were not included in our study since HIV-infected tuberculosis patients are usually not admitted to specialized tuberculosis hospitals in China, and also the study findings are only applied to adult patients since individuals younger than 15 years old were not included in this study. Fourth, the experimental data of clinical isolates in drug susceptibility test of pyrazinamide (PZA), an important anti-tuberculosis drug, is generally insufficient in clinical practice due to time-consuming and technically demanding. The low pH medium and the stringent inoculum size of the assays often lead to inconsistent and nonreproducible results which deter clinicians from relying on these tests for guidance. Hence those data were unavailable in this study[34]. Also, we should point out that demographic pattern and epidemiological link associated with drug resistance development are multifactorial. Some exogenous factors such as poor ventilation and poor-hygiene in overcrowded area and intrinsic resistance as a result of MDR-TB epidemic/outbreak were not discussed in this study.

To our knowledge, our research is the first large scale multi-hospital investigation to assess the drug resistant patterns of patients with MDR-TB in China. Our findings may represent MDR-TB clinical characteristic and current situation of drug resistance of MDR-TB in hospital settings in China. And we highlighted some concerns for MDR/XDR-TB control in China. Timely and routine DST is necessary to ensure an early detection of MDR-TB and its proper treatment. Additional assistance should be provided to vulnerable groups who cannot afford SLD in less developed areas to enhance the continuity of treatment and to raise awareness of proper TB treatment. The management of outpatients needs to be strengthened to ensure the treatment adherence. Rational use of drugs should be enshrined in policies and implemented.
